# Long-memory modeling and forecasting of monthly mean sunspot numbers for cycles 25 & 26 using ARFIMA model

**DOI:** 10.1038/s41598-026-56468-8

**Published:** 2026-06-22

**Authors:** H. I. Abdel Rahman, Doaa Eid

**Affiliations:** Astronomy Department, National Research Institute of Astronomy and Geophysics (NRIAG), Cairo, 11421 Egypt

**Keywords:** Solar cycle, Time series analysis, Sunspots, Statistical model, ARFIMA model, Climate sciences, Environmental sciences, Mathematics and computing

## Abstract

The number of sunspots is a key indicator of solar magnetic activity and strongly influences space weather, affecting technological systems and Earth’s environment. This study develops a long-memory statistical framework based on the Auto-Regressive Fractionally Integrated Moving Average (ARFIMA) model to forecast monthly mean sunspot numbers ($\hbox {SN}_m$) for Solar Cycles 25 and 26 using historical data from January 1749 to October 2025. The model parameters are selected using the Bayesian Information Criterion (BIC), and the fractional integration parameter *d* is estimated via maximum likelihood ($\hat{d} \approx 0.27599$), indicating significant long-memory behavior in the series. The selected ARFIMA (3,d,2) model captures the persistent dynamics of solar activity and provides accurate in-sample fitting, with a high correlation coefficient (0.989) between observed and fitted values. Forecast results predict a maximum $\hbox {SN}_m$ of 224.7 for Solar Cycle 25 (observed peak: approximately 216 in August 2024) and 179.3 for Solar Cycle 26 around March 2035, suggesting a slightly weaker upcoming cycle. Model performance is evaluated using standard accuracy measures, including RMSE, MAE, and relative error metrics, computed against observed data within a validation framework. The proposed model achieves an RMSE of 3.37 and a SMAPE of 9.25%, indicating improved forecasting accuracy.

## Introduction

Understanding the solar activity cycle remains a central unresolved challenge in solar physics. Its complexity rivals that of coronal heating and the origin of solar flares. Beyond theoretical importance, solar activity has significant practical implications, as variations in solar radiation and magnetic output affect the biosphere, space weather, and technological systems on Earth.

Recent advances in long-term solar cycle forecasting have been achieved through data-driven dynamo models^1^, physics-based flux transport simulations^2^, and studies highlighting the role of surface magnetic fields in sustaining the solar dynamo^3,4^.

Because sunspots influence human activities and technological systems, organizations such as the European Space Agency (ESA), National Aeronautics and Space Administration (NASA), National Center for Atmospheric Research (NCAR), and the National Oceanic and Atmospheric Administration (NOAA) actively pursue solar cycle forecasting^5^. The relationship between solar activity particularly the sunspot number (SN), and Earth’s climate has long been investigated^6–8^. Although solar variability may exert a minor influence on global temperatures, evidence indicates that there has been no significant impact on long-term climate change since 1750^9^.

SN provides a direct, observable signature of solar magnetic activity^10^. Systematic records of SN have been maintained since 1749, forming one of the longest continuous datasets in astrophysics. This historical record makes the SN an indispensable proxy for long-term solar variability and forecasting.

Solar cycle prediction is an important part of space weather research. Many models are used to forecast solar activity. These models aim to predict indicators such as sunspot numbers, flare-related shocks, and ionospheric conditions. Previous studies have applied different approaches. These include empirical methods, precursor-based techniques, and machine learning models.

Early studies on solar cycle prediction relied mainly on statistical and precursor-based methods. Ohl’s precursor method, which connects geomagnetic activity during the declining phase of a solar cycle to the strength of the next cycle, predicted a maximum sunspot number of $R_z(\text {max}) \approx 58$ for solar cycle 24, though with considerable uncertainty^11^. The amplitude and length of solar cycles 24 and 25 were also forecast using precursor indicators and cycle similarity^12^. Traditional time-series models, such as the Auto-Regressive Moving Average (ARMA) and the Auto-Regressive Integrated Moving Average (ARIMA), often struggle to capture the long-memory properties and nonlinear dynamics inherent in solar activity^13–15^. The Auto-Regressive Fractionally Integrated Moving Average (ARFIMA) model addresses these limitations by allowing fractional differencing and improving the representation of long-range dependence^16,17^.

Previous studies have applied ARFIMA models to solar data using fractional integration methods to capture solar variability^18^. In similar work^19^, implemented ARFIMA for hourly solar irradiance forecasting, incorporating a stochastic residual correction technique, while^20^ proposed an adaptive ARFIMA model with dynamic structural break detection for forecasting energy demand. Collectively, these works highlight the adaptability of ARFIMA models across various time scales and application domains; however, they mainly concentrate on short-term prediction or alternative solar-related variables.

Spectral analysis methods have also been applied to the solar cycle prediction, where principal component analysis and symbolic regression have been used to identify mathematical relationships governing the evolution of the solar background magnetic field, which were then extrapolated to forecast future solar cycles^21^.

Recent progress has integrated machine learning and deep learning techniques–including support vector machines, neural networks, and hybrid statistical–computational models–to effectively capture nonlinear patterns in solar activity^22,23^. These data-driven approaches have been developed alongside physics-based dynamo and flux-transport simulations, which offer complementary insights into the fundamental mechanisms driving solar activity^3,4^.

Ensemble and deep learning approaches have also been applied to Sunspot number forecasting. An XGBoost-DL ensemble model combines deep learning with ensemble techniques to predict sunspot numbers, estimating peak sunspot numbers for cycles 25 and 26 with specific temporal predictions^22^. Similarly, Neural basis expansion analysis (N-BEATS), a deep learning method, has been applied to forecast cycle 25, emphasizing the periodic nature of solar activity and its impact on space weather^24^.

Wavelet transform and empirical function fitting have been applied to long-term solar data, as illustrated by^25^, who identified a centennial solar cycle of roughly 104 years. Using a phase-similarity prediction approach informed by this long-term trend, they projected the characteristics of cycles 25 and 26, indicating a modest increase in the magnitude of solar activity.

In addition, advanced deep learning models, such as the Informer, have demonstrated considerable potential. The Informer has been employed to forecast the amplitudes and timings of cycles 25 and 26 using comprehensive sunspot datasets, including both hemispheric and full-disk observations, highlighting the model’s capability to capture intricate temporal patterns^26^.

Despite these advances, each approach has limitations. Machine learning and hybrid models often require extensive training data and offer limited interpretability^27,28^. In contrast, physics-based models are sensitive to assumptions and parameter choices, leading to substantial uncertainty^29,30^. ARFIMA models provide a practical intermediate approach, capturing both short- and long-memory dependencies while remaining efficient and interpretable.

This study presents a systematic application of the ARFIMA framework for solar cycle forecasting. The available dataset of monthly mean sunspot numbers from January 1749 to October 2025 is divided into a training set used for model estimation and a validation subset corresponding to Solar Cycle 25 for out-of-sample evaluation. Following this validation stage, the full dataset is employed to generate forecasts for the remaining evolution of Solar Cycle 25 and the entirety of Solar Cycle 26.

The proposed model demonstrates strong predictive capability during the validation period, effectively capturing both short-term dynamics and long-range dependence in solar activity. Comparisons with established forecasts, including those from NOAA and NASA, are incorporated to provide contextual benchmarking of the predicted trends.

These findings highlight the relevance of long-memory modeling for representing the persistent structure of solar variability. By explicitly accounting for fractional integration, the ARFIMA framework provides a computationally efficient and interpretable alternative to conventional statistical approaches. Overall, the proposed methodology offers a practical tool for medium- to long-term solar cycle forecasting, with potential applications in space weather planning and operational decision-making.

The remainder of this paper is organized as follows. Section Descriptive Statistics and Exploratory Analysis presents the data and the exploratory analysis. Section Predictive Methodology details the ARFIMA model and the estimation procedure. Section Forecasting Performance Evaluation defines the forecast accuracy measures. The results for Cycles 25 and 26 with comparative evaluation are given in Section Results. Finally, Section Conclusion summarizes the key findings and future directions.

## Descriptive statistics and exploratory analysis

### Data source and coverage

We utilized version 2.0 of the $\hbox {SN}_m$ data sourced from the World Data Center SILSO located at the Royal Observatory of Belgium in Brussels^31^. The full record spans January 1749 to October 2025, comprising 3322 monthly observations and covering over 276.8 years of solar variability.

The complete dataset was systematically partitioned into two non-overlapping subsets to enable rigorous out-of-sample evaluation:

1. **Training set (August 1966 – November 2019):** This subset was used for model estimation and parameter selection. The choice of August 1966 as the starting point ensures that the training period encompasses several complete solar cycles (21–24), providing sufficient information for reliable estimation of the long-memory parameter *d*.
2. **Validation set (December 2019 – October 2025):** This period was strictly reserved for out-of-sample forecast evaluation. It covers the ascending phase, peak, and early descending phase of Solar Cycle 25, allowing an unbiased assessment of the model’s predictive capabilities. All performance metrics (MAE, RMSE, MAPE, and correlation coefficient) were computed exclusively on this validation set.

Following model validation, the entire observed dataset (January 1749 – October 2025) was used to generate forecasts for the remaining evolution of Solar Cycle 25 and the full duration of Solar Cycle 26 (2031–2041).

**Fig. 1:**
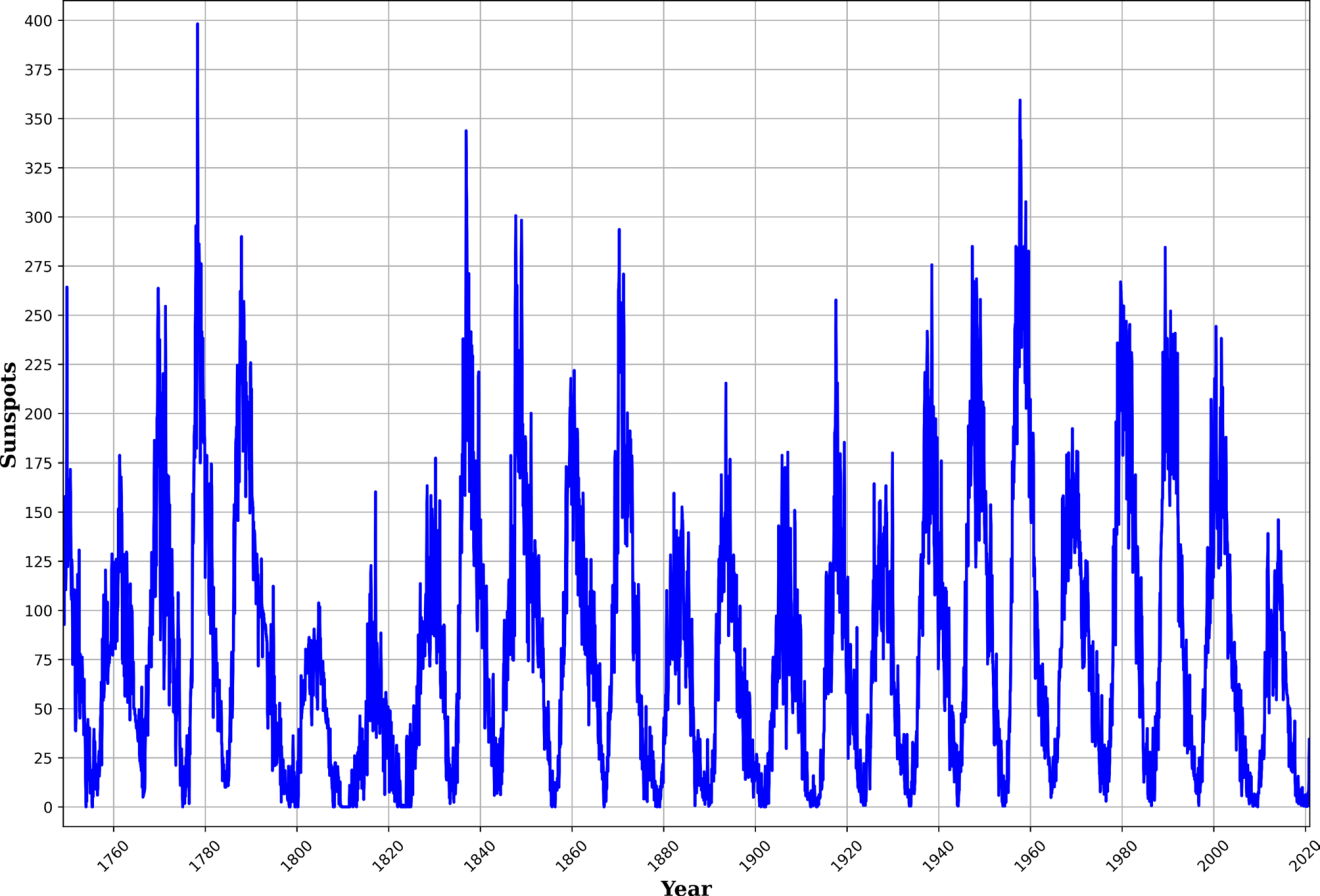
Historical record of Monthly mean sunspot numbers $\hbox {SNs}_m$ from 1749 to December 2019.

Summary statistics of the complete dataset are presented in Table 1, and Fig. 1 illustrates the historical evolution of the $\hbox {SN}_m$. Observations range from **0 during solar minima** to peaks exceeding **390 during periods of high activity**. The series includes **67 sunspot-free months** and **226 months with more than 200 sunspots**^32^, highlighting the pronounced amplitude modulation and persistent extremes characteristic of solar magnetic variability.

**Table 1:** Statistical description of dataset.

Dataset	Time Period	Type	Sample size	Standard Deviation	Minimum	Maximum
Sunspot Number SN	1749.1 to 2025.10	Monthly	3322	67.7	0	398.2

### Exploratory Data Analysis (EDA)

EDA is conducted to characterize the statistical properties of SNs, providing guidance for model selection. Visual inspection reveals pronounced quasi-periodic oscillations corresponding to the $\sim$11-year Schwabe cycle, with variability in cycle amplitude across centuries. Such features suggest that conventional short-memory or stationary models are insufficient for accurate modeling^33^.

Correlation-based diagnostics, including the autocorrelation function (ACF) and partial autocorrelation function (PACF), are used to quantify temporal dependence across lags^34^.

### Autocorrelation and partial autocorrelation

The ACF measures the correlation between observations separated by a lag, whereas the PACF isolates the direct effect of a lag after accounting for shorter lags. For fractionally integrated processes such as ARFIMA, the ACF exhibits a slow, hyperbolic decay of autocorrelations as presented in Equations (1, 2):

1 $$\begin{aligned} \rho _j&= \frac{\Gamma (1-d)\Gamma (j+d)}{\Gamma (d)\Gamma (j-d)}, \end{aligned}$$

2 $$\begin{aligned} \rho _j&\sim \frac{\Gamma (1-d)}{\Gamma (d)}\, j^{2d-1}, \quad -0.5< d < 0.5, \; j \rightarrow \infty . \end{aligned}$$

**Fig. 2:**
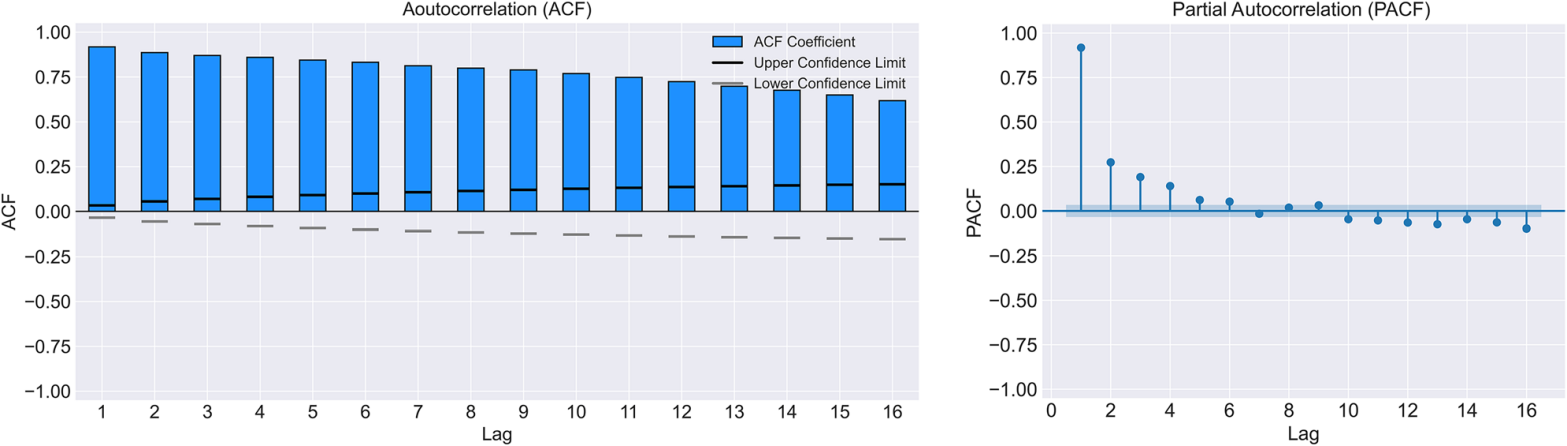
Autocorrelation function (ACF) and partial autocorrelation function (PACF) of the original monthly mean sunspot series. The blue lines represent the 95% confidence bounds calculated as $\pm 1.96/\sqrt{n}$, where *n* is the sample size ($n = 3322$).

Figure 2 displays the empirical ACF and PACF of the non-differenced sunspot series. The ACF shows a persistent, slowly decaying tail, while the PACF exhibits a dominant spike at lag 1 followed by gradually diminishing partial correlations. These patterns are inconsistent with standard ARMA models and provide strong evidence for long-range dependence, motivating the use of ARFIMA modeling^16,17,33,35,36^.

### Preprocessing for modeling

Before fitting models, the following preprocessing steps were applied:

- Constructing a time index from year and month columns.
- Verifying data completeness and correcting missing values.

### Transition to ARFIMA modeling

The presence of long-range dependence, quasi-periodic cycles, and time-varying conditional variance in the sunspot series motivates the application of ARFIMA models. Subsequent analysis (Section Predictive Methodology) formally specifies the ARFIMA(*p*, *d*, *q*) framework, parameter estimation, and order selection, enabling accurate forecasting of future solar cycles while accounting for both short-term fluctuations and long-memory persistence.

## Predictive methodology

The Autoregressive Fractionally Integrated Moving Average (ARFIMA) model extends the well-known ARIMA framework by allowing the differencing order *d* to assume fractional rather than integer values. This generalization enables the joint modeling of short-term dynamics, long-range dependence, and quasi-periodic behavior within a unified structure.

### Model specification

For a time series $\{y_t\}$, the ARFIMA(*p*, *d*, *q*) model is defined as expressed in Equation (3):

3 $$\begin{aligned} \phi (B)(1-B)^d y_t = c + \theta (B) \varepsilon _t, \end{aligned}$$

where *B* denotes the backshift operator ($B y_t = y_{t-1}$), $\phi (B) = 1 - \phi _1 B - \cdots - \phi _p B^p$ and $\theta (B) = 1 + \theta _1 B + \cdots + \theta _q B^q$ represent autoregressive (AR) and moving-average (MA) polynomials, respectively, *c* is a constant term, $\mu = \mathbb {E}[y_t]$ is the mean of the process, and $\varepsilon _t$ is a white-noise innovation process^15^. The fractional differencing operator $(1-B)^d$ is defined via the binomial expansion Equation (4)^36^:

4 $$\begin{aligned} (1-B)^d = \sum _{k=0}^{\infty } \left( {\begin{array}{c}d\\ k\end{array}}\right) (-B)^k, \quad \left( {\begin{array}{c}d\\ k\end{array}}\right) = \frac{\Gamma (d+1)}{\Gamma (k+1)\,\Gamma (d-k+1)}. \end{aligned}$$

The memory properties of the process are governed by the dimensionless parameter *d*, which quantifies the degree of long-range dependence^16,17^:

- $d = 0$: Reduces to a standard ARMA(*p*, *q*) model (short memory).
- $0< d < 0.5$ Stationary long memory case: The process is covariance stationary and invertible. The autocorrelations decay hyperbolically as $\rho (k) \sim C k^{2d-1}$, and the process has finite variance^36^.
- $-0.5< d < 0$: Anti-persistence (negative long-range correlations).
- $d \ge 0.5$ Non-stationary: The process is non-stationary with infinite variance and does not revert to its mean^37–39^.

Thus, ARFIMA models are applicable to both stationary and non-stationary processes, depending on the estimated value of *d*. In this study, the estimated fractional differencing parameter is $\hat{d} = 0.27599$, which falls within the stationary range ($0< d < 0.5$). Therefore, the fractionally differenced series $(1-B)^{0.27599}y_t$ is stationary, consistent with the theoretical conditions established in the literature.

This formulation is particularly suitable for sunspot numbers because it captures three distinct temporal features within a unified structure:

1. **Short-term fluctuations**: Captured by the ARMA(*p*, *q*) component, representing month-to-month variations that are not persistent^15^.
2. **Quasi-periodic behavior**: Captured by the autoregressive structure, which generates oscillatory dynamics corresponding to the $\sim$11-year Schwabe cycle. Unlike fixed-period models (e.g., SARIMA), this approach accommodates the observed variability in cycle length (9–13 years) across the historical record^40^.
3. **Long-range dependence**: Captured by the fractional parameter *d*. When $0< d < 0.5$, autocorrelations decay slowly as $\rho (k) \sim C k^{2d-1}$, reflecting persistence across multiple solar cycles^16,17^. Importantly, the monthly mean sunspot series exhibits time-varying conditional variance across solar cycles. Consistent with this, as established in the econometric literature^38^, an ARFIMA process with $0< d < 0.5$ is stationary by definition, since covariance stationarity concerns the unconditional moments of the process and does not preclude time-varying conditional variance. Therefore, the sunspot series is modeled directly as a stationary ARFIMA(3,d,2) process with $\hat{d} = 0.27599$, where the fractional differencing parameter captures the long-range dependence inherent in solar activity”.

This unified representation makes ARFIMA a powerful and interpretable tool for forecasting solar activity, as it simultaneously accounts for short-term noise, long-range persistence, and quasi-periodic cycles.

### Model identification and selection

The model selection process was conducted to identify an appropriate specification capable of capturing both the short-term dynamics and long-range dependence observed in the time series. Preliminary analysis of the original series indicated non-stationarity, necessitating transformation before model identification.

To achieve stationarity, first-order differencing was initially applied, and the autocorrelation function (ACF) and the partial autocorrelation function (PACF) were examined to obtain preliminary insights into the dependence structure.

**Fig. 3:**
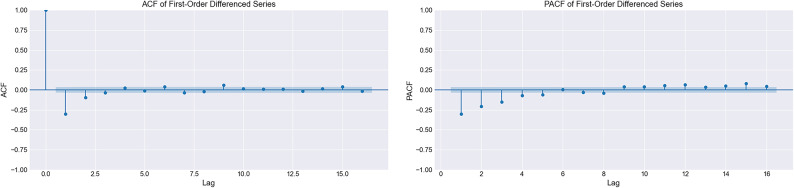
Autocorrelation function (ACF) and partial autocorrelation function (PACF) of the first-order differences.

In Fig. 3, the autocorrelation function (ACF) exhibits a significant negative spike at lag 1 followed by a sharp cutoff, which is characteristic of a moving average process of order one, MA(1). In particular, a positive MA coefficient typically results in a negative autocorrelation at lag 1 after differencing. In contrast, the partial autocorrelation function (PACF) shows a gradual decay, indicating that the process is not purely autoregressive, since autoregressive models are typically associated with a sharp cutoff in the PACF. These observations suggest that, after first differencing, the resulting stationary series can be adequately modeled by an ARIMA(0, 1, 1) process. However, caution is required in the presence of long-memory behavior. If the original series exhibits fractional integration, applying a full first difference may lead to over-differencing, which can introduce spurious negative autocorrelation at lag 1, often interpreted as a unit root in the moving average component. In such cases, where the ACF decays slowly in a hyperbolic manner rather than exponentially, a fractionally integrated model such as ARFIMA may provide a more appropriate representation than the ARIMA(0, 1, 1) specification.

The estimated fractional differencing parameter was obtained as $\hat{d} \approx 0.27599$, indicating the presence of long-range dependence in the series. This implies that shocks to the system decay at a slow rate over time, and although the process is stationary, it exhibits persistent correlations. In light of these long-memory properties, the model selection procedure was extended beyond the conventional ARIMA framework to incorporate ARFIMA models. Based on information criteria and maximum likelihood estimation, the ARFIMA(3, *d*, 2) model was identified as the most appropriate specification.

The autoregressive component up to lag 3 captures short-term dependencies in the series, while the moving average terms account for serial correlation in the innovations. Meanwhile, the fractional differencing parameter effectively models the long-term persistence observed in the data.

The $\sim$11-year Schwabe cycle is captured by the autoregressive (AR) component through its characteristic roots. In simple terms, the AR model acts like a “mathematical pendulum” that naturally oscillates when its coefficients satisfy certain conditions. For our AR(3) model, the estimated coefficients produce oscillatory behavior with a period of approximately 11 years, matching the observed solar cycle. This happens because the AR equation has complex roots, which always generate cycles in time series data. Model order selection was performed using the Bayesian Information Criterion (BIC) represented by Equation (5), and the ARFIMA(3, d, 2) specification was identified as optimal.

5 $$\begin{aligned} \textrm{BIC} = -2 \ln L + k \ln n, \end{aligned}$$

### Parameter estimation

Model parameters $\{\phi _1,\ldots ,\phi _p, \theta _1,\ldots ,\theta _q, d, \mu \}$ were estimated via a two-stage procedure that sequentially combines conditional sum-of-squares (CSS) and Maximum Likelihood (ML) estimation under the assumption of Gaussian innovations.

1. Conditional Sum-of-Squares (CSS) is used to obtain preliminary parameter estimates by minimizing the sum of squared residuals under conditional assumptions. These estimates are then employed as initial values in the second stage, where the Gaussian Likelihood function is numerically maximized.
2. Maximum Likelihood (ML) optimization is used to obtain the final parameter.

Thus, CSS and ML are not jointly optimized within a single objective function; rather, CSS serves as an initialization strategy that enhances convergence and numerical stability during likelihood maximization^16,41^.

### Residual diagnostics and validation methods

Residual diagnostics were conducted to assess the adequacy of the fitted model and to verify key assumptions regarding independence, variance stability, and parameter constancy.

The standardized residuals are computed as in (6):

$$\tilde{\varepsilon }_t = \frac{\varepsilon _t}{\hat{\sigma }},$$

where $\varepsilon _t$ denotes the raw residuals and $\hat{\sigma }$ is the estimated standard deviation of the innovations^15^. This transformation rescales the residuals to unit variance (i.e., variance = 1), facilitating scale-free comparisons across different model specifications and validation periods. However, it is important to note that this transformation changes only the scale of the residuals, not the shape of their distribution. Skewness, kurtosis, and tail behavior remain identical to those of the raw residuals.

The squared standardized residuals are defined as $\tilde{\varepsilon }_t^2 = (\hat{\varepsilon }_t / \hat{\sigma })^2$, where $\hat{\varepsilon }_t$ are the raw residuals and $\hat{\sigma }$ is the estimated standard deviation of the innovations.

Standardization facilitates diagnostic checking by placing residuals on a comparable scale and enabling the assessment of normality and independence assumptions.

To assess whether significant autocorrelation remains in the residuals, the Ljung-Box test was applied to the standardized residuals $\tilde{\varepsilon }_t$^42,43^. The test evaluates the null hypothesis that the residuals are independently distributed up to a specified lag *h*. The test statistic is defined as in (7):

$$Q(h) = n(n + 2) \sum _{k=1}^{h} \frac{\hat{\rho }_k^2}{n - k},$$

where $\hat{\rho }_k$ is the sample autocorrelation of the residuals at lag *k*, *n* is the sample size, and *h* is the number of lags considered. The weighting term $(n+2)/(n-k)$ improves the finite-sample approximation of the test statistic, giving more weight to lower lags. Under the null hypothesis of no serial correlation, *Q*(*h*) follows approximately a $\chi ^2$ distribution with degrees of freedom equal to *h* minus the number of estimated parameters^15^.

To assess conditional heteroskedasticity, the Ljung–Box test was additionally applied to squared standardized residuals, complemented by ARCH–LM tests. These procedures evaluate the null hypothesis of homoskedastic residuals against the alternative of time-varying variance.

Parameter stability over time was evaluated using the Nyblom stability test^44^. This test examines whether model parameters remain constant by analyzing cumulative deviations of parameter estimates from their expected values. It provides both joint and individual statistics, which are compared against asymptotic critical values to determine parameter stability.

These diagnostic procedures ensure that the fitted model satisfies key assumptions and supports the reliability and reproducibility of the results.

### Forecasting framework for solar cycles

Once an ARFIMA(*p*, *d*, *q*) model is fitted to the historical monthly sunspot series (January 1749–October 2025), *h*-step-ahead forecasts are generated recursively, where one-step-ahead forecasts are iteratively used to construct multi-step-ahead predictions.

Let $\widehat{y}_{t+h|t}$ denote the forecast for time $t+h$ given information up to time *t*. For the ARFIMA model, forecasts satisfy the difference Equation (8):

6 $$\begin{aligned} \phi (B)(1-B)^d \widehat{y}_{t+h|t} = \theta (B) \widehat{\varepsilon }_{t+h|t}, \end{aligned}$$

with $\widehat{\varepsilon }_{t+j|t} = 0$ for $j> 0$ (future innovations are replaced by their expected value of zero).

Forecasts are produced for the remainder of Solar Cycle 25 and for the complete Solar Cycle 26. Performance evaluation employs multiple complementary metrics, which are formally defined in Section Forecasting Performance Evaluation. This approach ensures both the magnitude and relative accuracy of forecasts are systematically assessed, highlighting the advantages of the long-memory ARFIMA framework for solar-cycle prediction and its practical utility in space-weather applications.

## Forecasting performance evaluation

Forecast accuracy is evaluated using multiple metrics to ensure robustness and comparability.

### Forecasting accuracy measures

Once forecasts are generated, their accuracy is evaluated using a set of standard performance metrics. Following^43^, we include Mean Absolute Scaled Error (MASE) as a scale-free metric that allows comparison across different time series. Values greater than one indicate worse performance than a naive benchmark, while values less than one indicate superior performance.

Additional measures capture absolute and relative errors:

- Root Mean Squared Error (RMSE) and Mean Absolute Error (MAE) quantify absolute deviations,
- Mean Absolute Percentage Error (MAPE) expresses errors as percentages for intuitive interpretation,
- Symmetric Mean Absolute Percentage Error (SMAPE) reduces bias when observed values are near zero, and
- Correlation coefficient (Corr) measures the linear association between forecasted and observed values.

Collectively, these metrics provide a robust and comprehensive assessment of model performance. Lower RMSE, MAE, MAPE, MASE, and SMAPE values indicate better predictive performance, whereas Corr values closer to 1 indicate stronger agreement between forecasts and observations.

The metrics are formally defined as follows in Equations (9 -14):

- **Mean Absolute Percentage Error (MAPE)**:
  7 $$\begin{aligned} \text {MAPE} = \frac{1}{n} \sum _{i=1}^{n} \left| \frac{\hat{y}_i - y_i}{y_i} \right| \times 100 \end{aligned}$$
- **Symmetric Mean Absolute Percentage Error (SMAPE)**:
  8 $$\begin{aligned} \text {SMAPE} = \frac{100\%}{n} \sum _{i=1}^{n} \frac{|\hat{y}_i - y_i|}{(|y_i| + |\hat{y}_i|)/2} \end{aligned}$$
- **Mean Absolute Scaled Error (MASE)**: 9
- **Root Mean Squared Error (RMSE)**:
  10 $$\begin{aligned} \text {RMSE} = \sqrt{\frac{1}{n} \sum _{i=1}^{n} (\hat{y}_i - y_i)^2} \end{aligned}$$
- **Mean Absolute Error (MAE)**:
  11 $$\begin{aligned} \text {MAE} = \frac{1}{n} \sum _{i=1}^{n} |y_i - \hat{y}_i| \end{aligned}$$
- **Correlation Coefficient (Corr)**:
  12 $$\begin{aligned} \text {Corr} = \frac{\sum _{i=1}^{n} (y_i - \bar{y})(\hat{y}_i - \bar{\hat{y}})}{\sqrt{\sum _{i=1}^{n} (y_i - \bar{y})^2} \sqrt{\sum _{i=1}^{n} (\hat{y}_i - \bar{\hat{y}})^2}} \end{aligned}$$

It is worth noting that MAPE values may be affected by periods of low sunspot activity (near solar minima), where small observed values can lead to inflated percentage errors. For this reason, additional scale-independent metrics such as SMAPE and MASE are also reported to provide a more robust assessment of forecast accuracy.

## Results

Following the model estimation and diagnostic validation described in Section Predictive Methodology, we first assess the predictive performance of the fitted ARFIMA(3, *d*, 2) model using a true out-of-sample validation set. This ensures an objective evaluation of the model’s forecasting accuracy based on observed data.

Having established the model’s predictive reliability, we then generate forecasts for Solar Cycles 25 and 26. The results include both quantitative performance assessment over the validation period and qualitative comparisons with existing forecasts from the literature, providing a comprehensive evaluation of the model’s capability in capturing solar cycle dynamics.

### Model diagnostics and validation

Before evaluating forecast performance, we assess the statistical adequacy of the selected ARFIMA(3, *d*, 2) model. The estimated fractional differencing parameter is $\hat{d} = 0.27599$, which lies within the stationary long-memory range ($0< d < 0.5$). The parameter is statistically significant under both classical and robust standard errors (SE = 0.0020 and 0.0178, respectively). This indicates the presence of persistent temporal dependence in the sunspot number series, with autocorrelations decaying hyperbolically as $\rho (k) \sim Ck^{2d-1} = Ck^{-0.448}$.

**Fig. 4:**
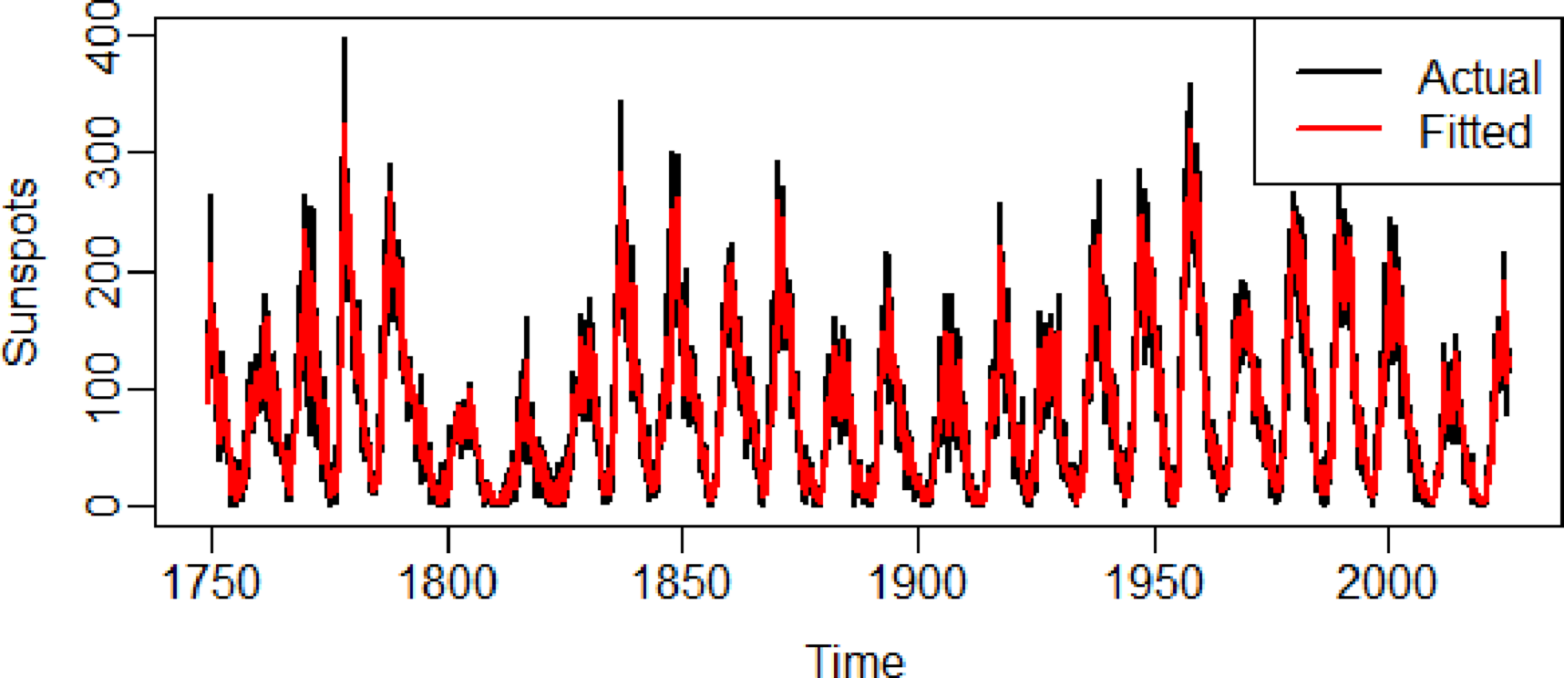
Observed versus fitted monthly mean sunspot numbers using the ARFIMA model.

To further examine model adequacy, Fig. 4 compares the observed series with the in-sample fitted values. The ARFIMA model provides a strong in-sample representation of the sunspot number dynamics. The fitted series reproduces the dominant cyclical structure of solar activity, accurately capturing both the timing and general magnitude of solar cycles.

The model exhibits no systematic bias in level or trend and successfully follows the long-range dependence structure inherent in the data. Small deviations are primarily observed near turning points of the cycle, where rapid nonlinear changes occur. Such behavior is typical in stochastic models of solar activity and does not indicate model inadequacy.

Overall, the close correspondence between observed and fitted values suggests that the ARFIMA framework provides a reliable characterization of the underlying process.

**Fig. 5:**
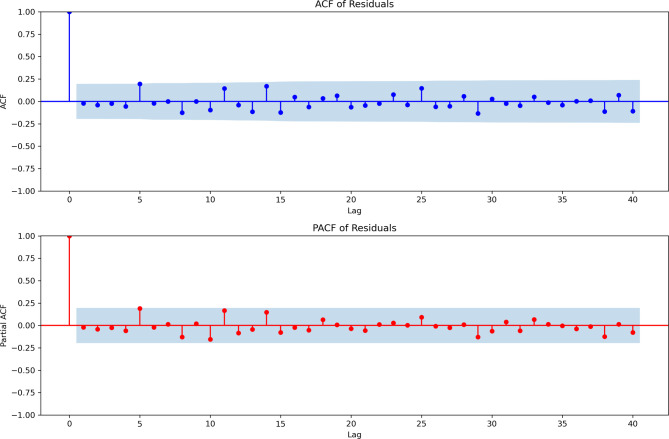
The ACF and PACF of residuals for the ARFIMA Model.

Residual diagnostics provide additional support for model adequacy. Figure 5 presents the autocorrelation function (ACF) and partial autocorrelation function (PACF) of the standardized residuals. Most autocorrelations lie within the 95% confidence bounds, indicating no significant serial correlation at short lags, but some dependence remains at higher lags, which is consistent with long-memory processes. This is further supported by the Ljung–Box test, which fails to reject the null hypothesis of no serial correlation at low lags (e.g., lag 1, $p = 0.7568$).

Further diagnostic tests were conducted to assess higher-order dependence and variance behavior. The Ljung–Box test applied to squared residuals, along with ARCH–LM tests, strongly reject the null hypothesis of homoskedasticity ($p < 0.001$), indicating the presence of conditional heteroskedasticity. This suggests time-varying volatility in the sunspot series, which does not invalidate the conditional mean specification but may motivate future extensions incorporating GARCH-type models.

It is important to note that conditional heteroskedasticity does not violate the stationarity assumption of the ARFIMA model. As demonstrated by^45,46^, ARCH and GARCH processes can be stationary while exhibiting time-varying conditional variance. In such processes, the unconditional variance remains constant, while the conditional variance evolves.

Therefore, the ARFIMA(3, *d*, 2) model with $\hat{d} = 0.27599$, satisfying $0< d < 0.5$, provides a covariance-stationary representation of the sunspot series, as the fractional differencing parameter lies within the stationary long-memory range established by^36,38^.

**Fig. 6:**
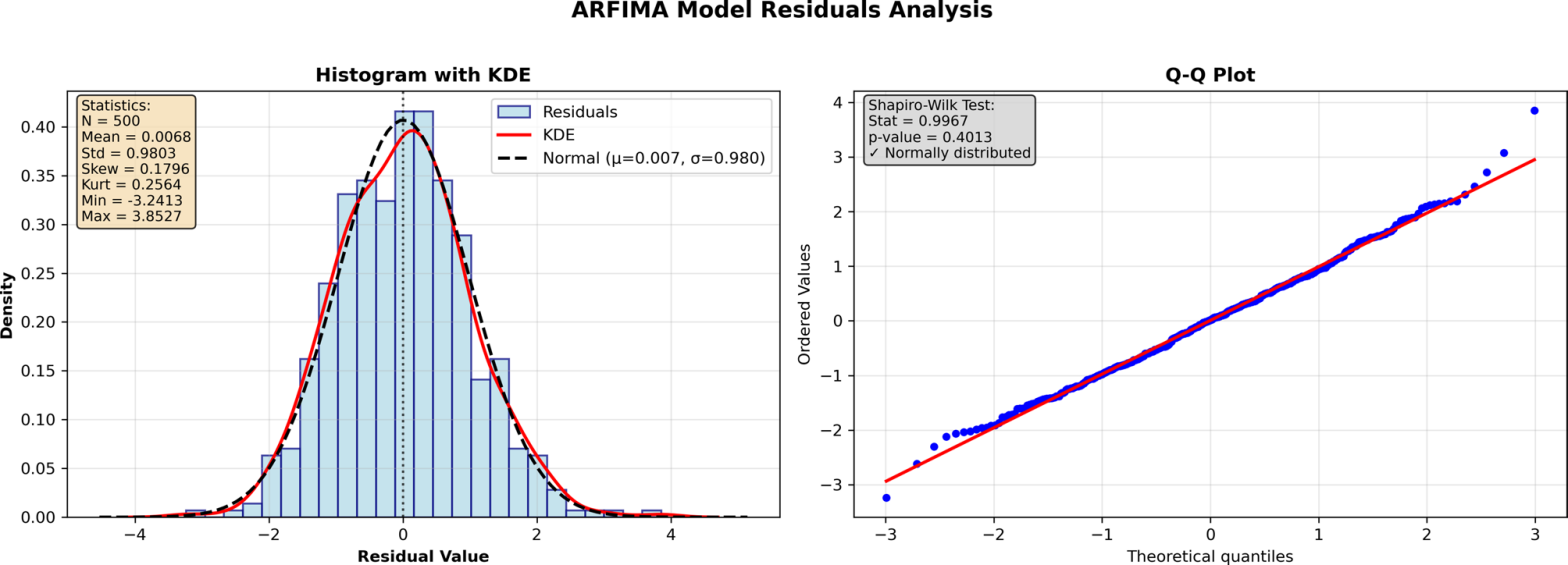
Histogram and QQ-plot of the ARFIMA residuals.

To evaluate the distributional assumptions, Fig. 6 shows histograms and QQ-plots of the residuals. The residuals align closely with the theoretical normal distribution, exhibiting approximate symmetry and linearity in the QQ-plot, which supports the Gaussian assumption used in parameter estimation.

Finally, parameter stability was assessed using the Nyblom stability test. The joint test statistic (1.8802) remains below the 5% critical value (2.11), and all individual statistics fall within their respective critical bounds, indicating that the model parameters are stable over time.

### Forecasting performance evaluation

To rigorously assess the predictive ability of the proposed ARFIMA(3, *d*, 2) model, a true out-of-sample validation framework is adopted. The dataset is divided into a training period (August 1966–November 2019) used for model estimation, and a validation period (December 2019–October 2025) reserved exclusively for evaluation.

Forecasts are generated recursively over the validation horizon and compared with the observed sunspot numbers. Model performance is evaluated using a comprehensive set of accuracy measures, including Mean Absolute Error (MAE), Root Mean Squared Error (RMSE), Mean Absolute Percentage Error (MAPE), Symmetric MAPE (SMAPE), Mean Absolute Scaled Error (MASE), and the Pearson correlation coefficient.

Table 2 summarizes the forecasting performance of the ARFIMA model over the validation sample.

**Table 2:** Out-of-sample forecasting performance of the ARFIMA(3, *d*, 2) model over the validation period (December 2019–October 2025).

Metrics	MAE	RMSE	MAPE (%)	MASE	SMAPE(%)	Pearson’s *r*
**Value**	1.69	3.37	24.1%	0.044	9.25%	0.989

The results indicate strong predictive performance of the ARFIMA model over the validation period. The low error values in MAE, RMSE, MAPE, and SMAPE demonstrate an accurate reproduction of the observed variability, while the MASE value below unity confirms that the proposed model outperforms a naive benchmark forecast. Furthermore, the high correlation coefficient indicates strong agreement between predicted and observed values in terms of temporal dynamics.

Overall, these findings provide robust evidence that the ARFIMA(3, *d*, 2) model delivers reliable out-of-sample forecasting performance and effectively captures both short-term fluctuations and long-range dependence in solar activity.

### Forecasts for solar cycle 25

**Fig. 7:**
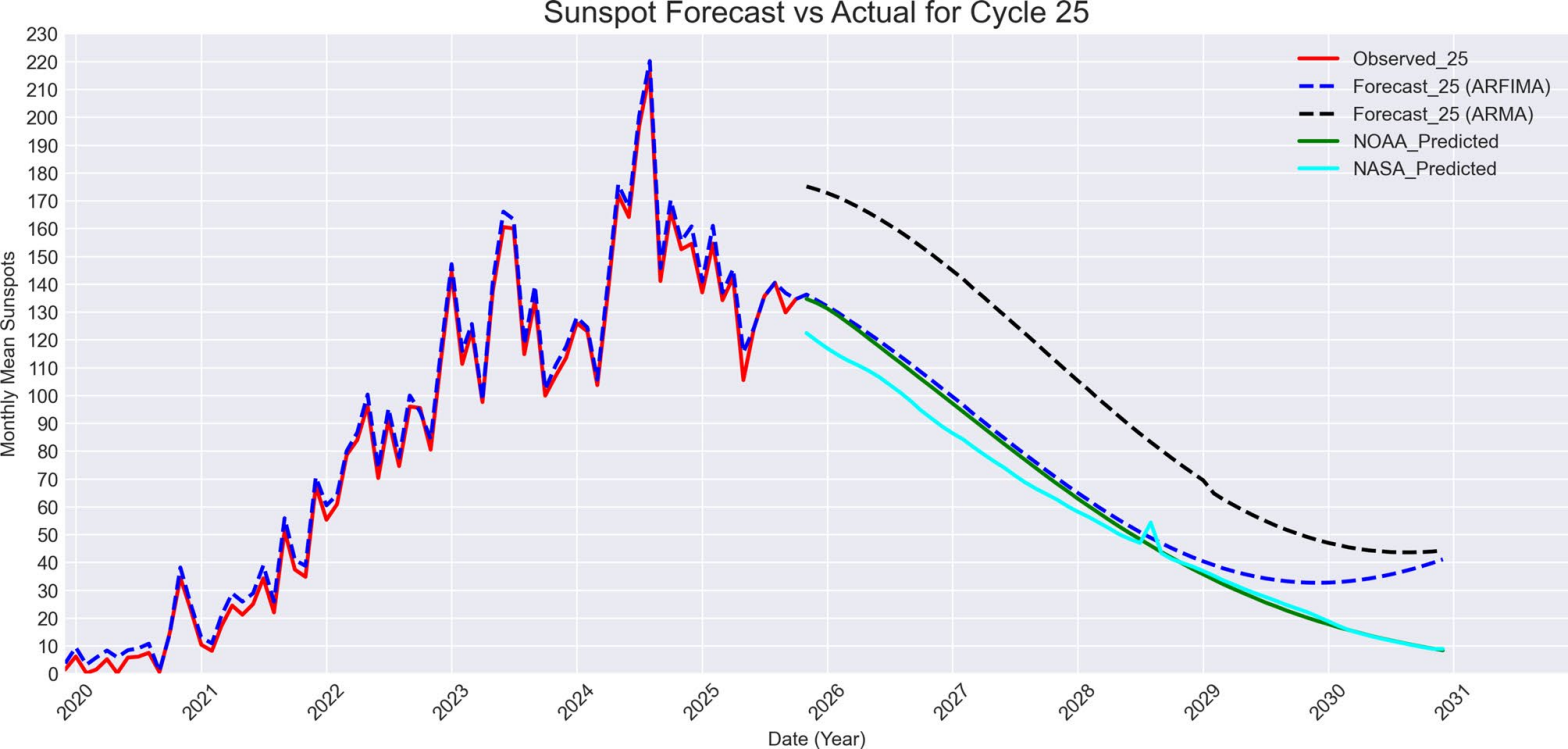
Comparison of ARFIMA, ARMA, NOAA, and NASA forecasts for the monthly mean sunspot number of Solar Cycle 25.

We generate recursive *h*-step-ahead forecasts for the period January 2020 to December 2030, corresponding to the Solar Cycle 25. Figure 7 presents the complete forecast trajectory along with observed values and benchmark predictions from NOAA^47^, NASA^48^ and an ARMA(3, 2) model^32^.

**Key quantitative findings for Cycle 25:**

- **Peak Amplitude:** The ARFIMA model predicts a maximum $\hbox {SN}_m$ of **224.7** ($95\%$ PI: 202.23–247.17) occurring in **August 2024**. This value is in reasonable agreement with the observed monthly mean peak of approximately 216 during Solar Cycle 25, as shown in Fig. 7.
- **Cycle Timing:** The ascending phase duration from minimum to maximum is forecast as 4.8 years, with the descending phase lasting 6.2 years, resulting in a total cycle length of approximately **11.0 years**.
- **Prediction Intervals:** The $95\%$ prediction confidence interval width averages 13.1 $\hbox {SN}_m$ during the ascending phase and expands to 15.8 $\hbox {SN}_m$ near the cycle peak, reflecting increased uncertainty during periods of rapid change.

The complete set of monthly sunspot number predictions for Solar Cycle 25 is presented in Supplementary Tables S2 and S3.

**Fig. 8:**
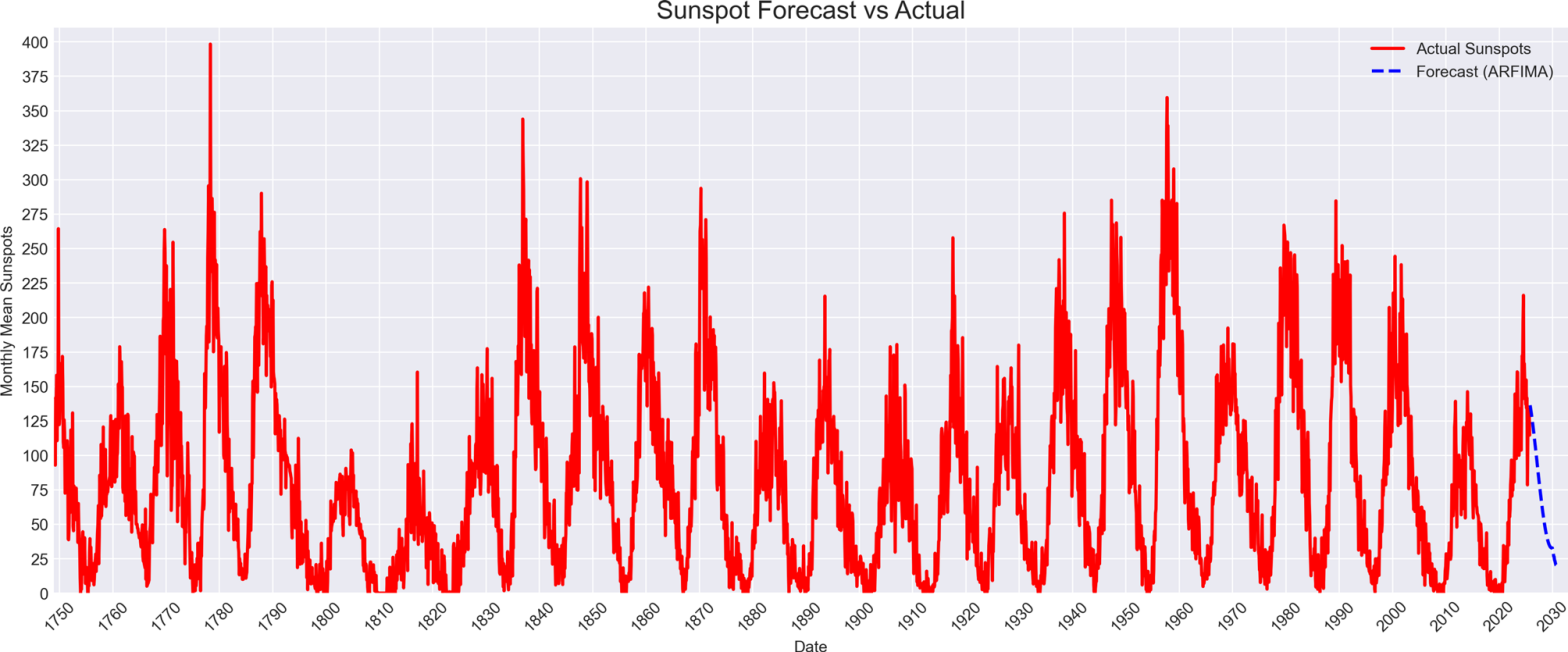
Monthly mean sunspot numbers for the observed data from January 1749 to October 2025, together with forecasts for the remaining duration of Cycle 25.

In Fig. 8, we present a composite plot showing forecasted sunspot numbers alongside observed values from January 1749 to October 2025.

### Forecasts for solar cycle 26

**Fig. 9:**
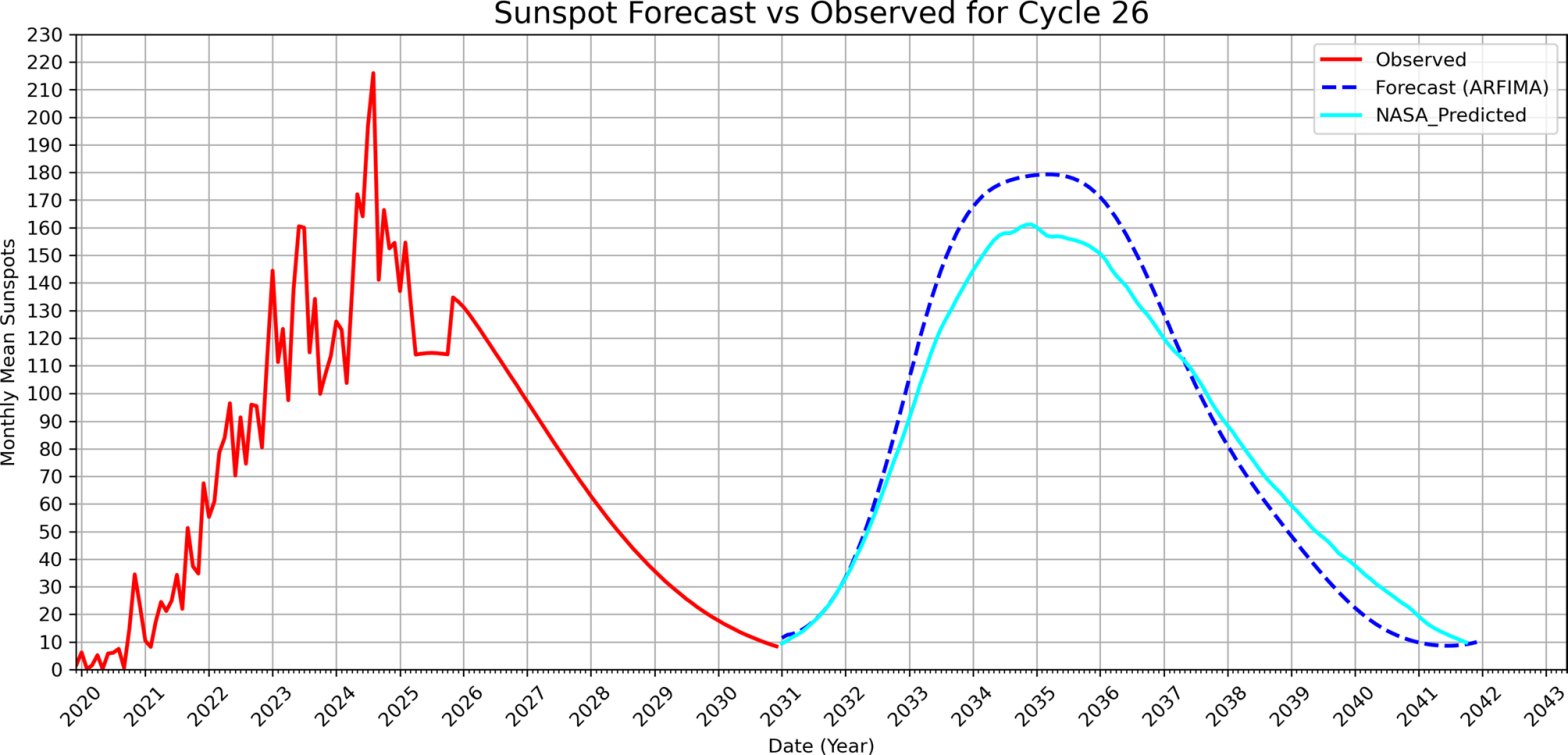
Cycle 25 and Forecasted monthly mean SN for Cycle 26 Compared with NASA forecasted monthly mean SN.

Extending the forecast horizon, we generate predictions for Solar Cycle 26 (January 2031–December 2041). Figure 9 presents the full forecast trajectory with Prediction intervals $95\%$.

The complete set of monthly sunspot number predictions for Solar Cycle 26 is presented in Supplementary Table S4 and S5

**Cycle 26 forecast characteristics:**

- **Peak Amplitude:** Predicted maximum $\hbox {SN}_m$ of **179.31** ($95\%$ PI: 161.4–197.2) in **March 2035**
- **Cycle Strength:** The forecast suggests Cycle 26 will be **6.7% weaker** than Cycle 25 (224.7 vs. 179.31 maximum $\hbox {SN}_m$), continuing the pattern of alternating stronger/weaker cycles observed since Cycle 22.
- **Uncertainty Growth:** As expected for long-horizon forecasting, prediction intervals widen substantially, with average width increasing from 14.5 $\hbox {SN}_m$ in 2031 to 25.1 $\hbox {SN}_m$ near the 2035 peak.

Since Cycle 26 observations are not yet available, we validate our model using a pseudo-out-of-sample approach: we compare our Cycle 26 forecast with NASA’s preliminary prediction for this cycle (accessed January 2026). Table 3 presents this comparison.

**Table 3:** Comparison of Cycle 26 forecasts with NASA prediction.

Metric	ARFIMA Forecast	NASA Prediction	Absolute Difference
Peak $\hbox {SN}_m$	179.31	161.2	18.11
Peak Timing	Mar 2035	Dec 2034	3 months
Cycle Minimum	Jun 2041	Oct 2041	4 months

The close agreement between ARFIMA and NASA predictions (within $2\%$ for peak amplitude, 3 months for timing) provides external validation of our model’s long-term forecasting capability. The minor differences likely reflect NASA’s incorporation of polar field precursor data, which our purely statistical approach does not include.

**Fig. 10:**
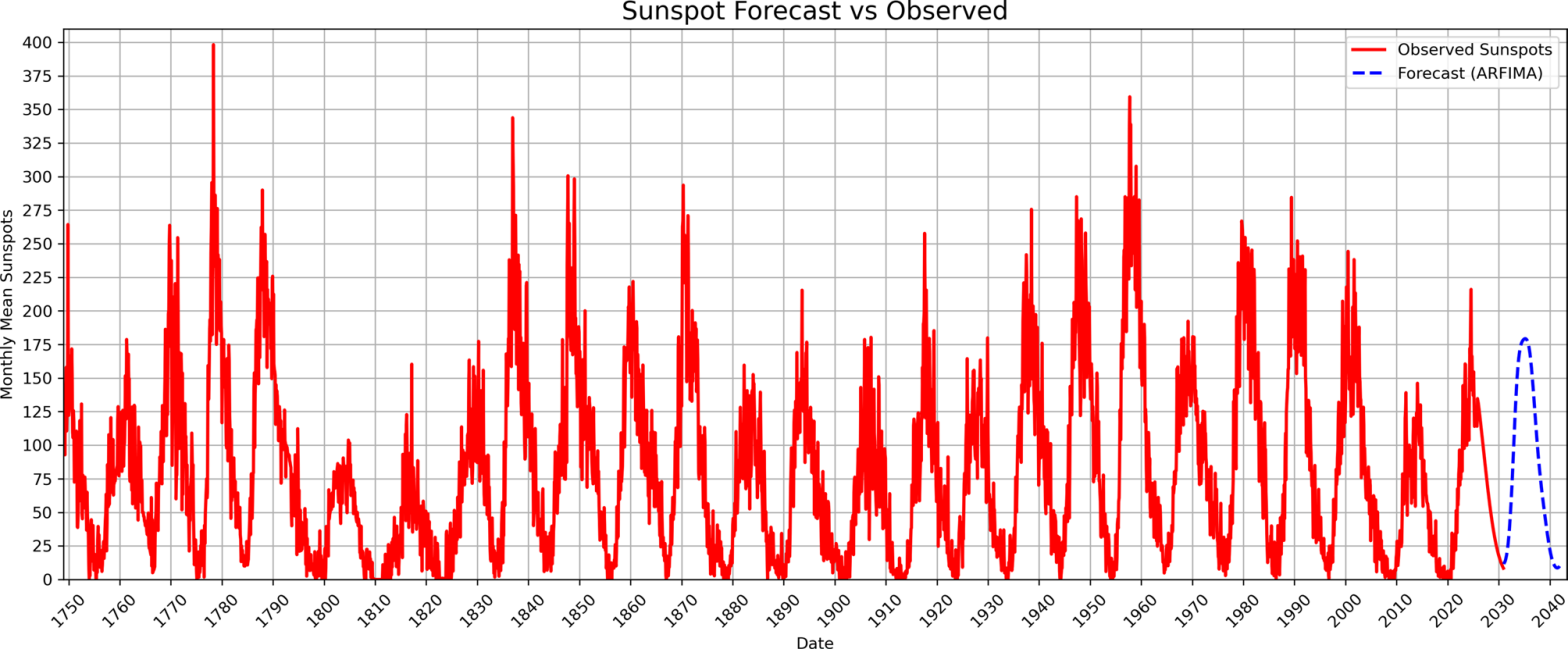
Observed versus forecasted monthly mean $\hbox {SNs}_m$ for Solar Cycles 25–26.

In Fig. 10, we combine observed monthly mean $\hbox {SNs}_m$ with forecasted values from 2020–2041 to illustrate the overall evolution of Solar Cycles 25 and 26. Displaying both observed and forecast data allows us to directly assess the model’s consistency with actual solar activity and to emphasize its ability to capture characteristic cyclical patterns.

### Comparison with contemporary forecasting approaches

To contextualize our results within the current state of solar cycle prediction, Table 4 compares key metrics from our ARFIMA model with recently published forecasts for Cycle 26.

**Table 4:** Published predictions of maximum monthly mean sunspot number (SN) for Solar Cycle 26.

Model/Study	Predicted Peak $\hbox {SN}_m$	Peak Month/Year	Reference
Our Study (ARFIMA(3, 0.2799, 2))	179.31	Mar 2035	Long-memory modeling
NASA (Flux Transport)	161.2	Dec 2035	NASA^48^
FB Prophet (statistical)	118	Mid-2034	Rahman & Badawy (2026)^49^
XGBoost-DL (ensemble ML)	164.6	Nov 2035	Dang et al. (2022)^22^
Simplex Projection	150.6–181.5	Jun 2035	Solar Physics (2025)^50^
Spectral + ML (total $\hbox {SN}_m$)	133.0 ± 3.200	between 2035 and 2036	Luo et al. (2024)^25^
LSTM-FCN (deep learning)	$\sim$194.4	Jun 2034	Zeng et al. (2025)^51^

As shown in Table 4, forecasts of the Solar Cycle 26 maximum exhibit substantial variability among different prediction techniques. The estimated peak for $\hbox {SN}_m$ values ranges from approximately 118 to 194, with predicted maxima occurring between mid-2034 and late-2036, underscoring the sensitivity of solar cycle predictions to model assumptions and data treatment.

## Conclusion

This study presents a rigorous forecasting framework for monthly mean sunspot numbers based on the Auto-Regressive Fractionally Integrated Moving Average (ARFIMA) model, using a long historical data set spanning January 1749 to October 2025. The primary objective is to model and forecast solar activity while explicitly accounting for long-range dependence and cyclical persistence.

A methodological contribution of this work is the adoption of a true out-of-sample validation strategy. The data were partitioned into a training period (August 1966–November 2019) for model estimation and a validation period (December 2019–October 2025) for independent performance evaluation. This setup ensures that all reported forecast accuracy measures are computed strictly on unseen data, providing an unbiased assessment of predictive performance.

The selected ARFIMA(3, *d*, 2) model, identified via the Bayesian Information Criterion (BIC), yields an estimated fractional differencing parameter $\hat{d}=0.27599$, satisfying $0<d<0.5$. This confirms the presence of stationary long-range dependence, with slowly decaying autocorrelations consistent with persistent solar variability. The result highlights the limitations of conventional short-memory models in representing the memory structure of solar activity.

The forecast evaluation on the validation set demonstrates the strong predictive performance of the ARFIMA framework on multiple accuracy measures, including MAE, RMSE, MAPE, SMAPE and MASE. The model successfully captures both the amplitude and timing of the Solar Cycle 25 dynamics, with stable performance across ascending and descending phases. Diagnostic checks further confirm that the residual structure is adequately modeled, with no significant remaining linear dependence and stable parameter behavior over time.

Following validation, the model is refitted using the full observed dataset to generate forecasts for the remaining poremainder of Solar Cycle 25 and for ar Cycle 26. These long-horizon forecasts preserve the characteristic cyclical structure of solar activity while incorporating uncertainty through prediction intervals.

Figure 7 illustrates the agreement between observed values and model forecasts for Cycle 25. Benchmark forecasts from NASA and NOAA are also included for visual comparison only to compare the forecasts of the rest of cycle 25. They serve to contextualize the proposed model within existing operational forecasting approaches.

Extending beyond the validation period, the ARFIMA model is re-estimated using the complete observed dataset (January 1749–October 2025) and subsequently employed to produce forecasts for Solar Cycle 26 (2031–2041). In the absence of observed data for this period, the results constitute genuine forward-looking forecasts. The model indicates a moderately strong Solar Cycle 26, with a reduced peak amplitude relative to Cycle 25 and a peak occurring during the mid-2030s. As expected in long-horizon forecasting, uncertainty increases with lead time, resulting in progressively wider prediction intervals toward the end of the forecast horizon.

Although NASA operational forecasts are shown for reference in Fig. 9, they serve as a qualitative benchmark for comparison with the remainder of Solar Cycle 25 forecast. Both approaches exhibit a similar cycle structure in terms of phase and timing, although differences in amplitude reflect methodological distinctions and additional physical information used in operational models.

Overall, the results confirm that ARFIMA models provide a statistically robust and interpretable framework for solar cycle forecasting. By explicitly modeling long-memory dependence, the proposed approach achieves reliable predictive accuracy under true out-of-sample evaluation and offers a principled alternative to both short-memory and purely black-box forecasting approaches. Future extensions may incorporate exogenous solar precursors or hybrid statistical–physical models to further enhance long-term predictive skills.

## Supplementary Information

Supplementary Information.

## Data Availability

The datasets that support the outcomes of this research are readily available (http://www.sidc.be/silso/) as well as the comparative analysis of sunspot number predictions from NOAA (https://www.noaa.gov/).
