# Supplementary material for: Long-memory modeling and forecasting of monthly mean sunspot numbers for cycles 25 & 26 using ARFIMA model
Source: Sci Rep. 2026 Jun 22;16:19381. doi: 10.1038/s41598-026-56468-8 (PMC13287477; doi:10.1038/s41598-026-56468-8)
Supplement: Supplementary file 1 — Supplementary Information. [file 41598_2026_56468_MOESM1_ESM.pdf]

Table S1. Estimated parameters of the ARFIMA$(3, d, 2)$ model.

Component	Parameter	Estimate	Std. Error	t-value	p-value
AR	$\hat{\phi}_1$	2.11962	0.000052	40576.00	<0.001
	$\hat{\phi}_2$	-1.25415	0.000402	-3120.50	<0.001
	$\hat{\phi}_3$	0.13232	0.000297	445.81	<0.001
MA	$\hat{\theta}_1$	-1.87287	0.000024	-79567.00	<0.001
	$\hat{\theta}_2$	0.87909	0.000000	20729000.00	<0.001
FI	$\hat{d}$	0.27599	0.002007[a]	137.55	<0.001
			0.017807[b]	15.50	<0.001
Mean	$\hat{\mu}$	93.02353	1.737590[a]	53.54	<0.001
			5.068950[b]	18.35	<0.001
Innovation Std.	$\hat{\sigma}$	24.42350	0.193237[a]	126.39	<0.001
			0.432950[b]	56.41	<0.001
Log-Likelihood		−15329.34			
AIC		9.2338			
BIC		9.2485			

[a]Classical standard error. [b]Robust standard error.

AR = Autoregressive; MA = Moving Average; FI = Fractional Integration.

The estimated $\hat{d} = 0.27599$ satisfies $0 < d < 0.5$, confirming covariance stationarity with long-range dependence.

Table S2. Monthly forecasted sunspot numbers for Solar Cycle 25 obtained using the AutoARFIMA model (2019-2024).

Year	Month	Forecast	Year	Month	Forecast	Year	Month	Forecast
2019	12	3.70	2021	7	38.90	2023	2	115.98
2020	1	9.44	2021	8	25.90	2023	3	125.67
2020	2	3.24	2021	9	55.87	2023	4	98.24
2020	3	5.81	2021	10	40.83	2023	5	140.69
2020	4	8.33	2021	11	38.75	2023	6	166.04
2020	5	5.87	2021	12	70.64	2023	7	163.26
2020	6	8.45	2022	1	60.49	2023	8	118.36
2020	7	9.09	2022	2	64.28	2023	9	139.34
2020	8	10.78	2022	3	80.02	2023	10	102.20
2020	9	0.92	2022	4	86.70	2023	11	110.94
2020	10	14.31	2022	5	100.31	2023	12	117.56
2020	11	38.16	2022	6	73.85	2024	1	128.05
2020	12	25.04	2022	7	95.31	2024	2	124.42
2021	1	12.96	2022	8	77.69	2024	3	105.67
2021	2	10.91	2022	9	99.98	2024	4	140.80
2021	3	20.89	2022	10	94.18	2024	5	175.82
2021	4	28.88	2022	11	84.28	2024	6	167.71
2021	5	25.89	2022	12	116.29	2024	7	200.49
2021	6	28.90	2023	1	147.19	2024	8	220.16

Table S3. Monthly forecasted sunspot numbers for Solar Cycle 25 obtained using the AutoARFIMA model (2024-2030).

Year	Month	Forecast	Year	Month	Forecast	Year	Month	Forecast
2024	9	145.72	2026	5	122.29	2028	1	64.99
2024	10	170.17	2026	6	119.63	2028	2	62.42
2024	11	155.52	2026	7	116.91	2028	3	59.93
2024	12	160.76	2026	8	114.12	2028	4	57.53
2025	1	140.91	2026	9	111.28	2028	5	55.21
2025	2	160.96	2026	10	108.40	2028	6	52.98
2025	3	136.92	2026	11	105.47	2028	7	50.86
2025	4	145.40	2026	12	102.52	2028	8	48.83
2025	5	115.50	2027	1	99.55	2028	9	46.91
2025	6	124.30	2027	2	96.56	2028	10	45.11
2025	7	135.60	2027	3	93.56	2028	11	43.41
2025	8	140.50	2027	4	90.57	2028	12	41.84
2025	9	136.80	2027	5	87.59	2029	1	40.38
2025	10	134.60	2027	6	84.63	2029	2	39.04
2025	11	136.25	2027	7	81.69	2029	3	37.83
2025	12	134.21	2027	8	78.79	2029	4	36.74
2026	1	132.03	2027	9	75.92	2029	5	35.78
2026	2	129.75	2027	10	73.10	2029	6	34.95
2026	3	127.35	2027	11	70.34	2029	7	34.25
2026	4	124.86	2027	12	67.63	2029	8	33.68
2029	9	33.23	2030	2	32.92	2030	7	35.65
2029	10	32.91	2030	3	33.23	2030	8	36.54
2029	11	32.73	2030	4	33.66	2030	9	37.53
2029	12	32.66	2030	5	34.21	2030	10	38.62
2030	1	32.73	2030	6	34.88	2030	11	39.81
						2030	12	41.10

Table S4. Monthly forecasted sunspot numbers for Solar Cycle 26 obtained using the ARFIMA model.

Year	Month	Forecast	Year	Month	Forecast	Year	Month	Forecast
2031	1	11.44	2033	1	105.48	2035	1	179.09
2031	2	12.56	2033	2	112.67	2035	2	179.24
2031	3	12.87	2033	3	119.68	2035	3	179.31
2031	4	13.58	2033	4	126.43	2035	4	179.28
2031	5	14.60	2033	5	132.85	2035	5	179.14
2031	6	15.89	2033	6	138.87	2035	6	178.86
2031	7	17.45	2033	7	144.45	2035	7	178.42
2031	8	19.29	2033	8	149.54	2035	8	177.79
2031	9	21.42	2033	9	154.13	2035	9	176.95
2031	10	23.86	2033	10	158.22	2035	10	175.88
2031	11	26.65	2033	11	161.81	2035	11	174.55
2031	12	29.81	2033	12	164.92	2035	12	172.95
2032	1	33.36	2034	1	167.58	2036	1	171.06
2032	2	37.32	2034	2	169.83	2036	2	168.87
2032	3	41.71	2034	3	171.71	2036	3	166.38
2032	4	46.54	2034	4	173.27	2036	4	163.60
2032	5	51.80	2034	5	174.56	2036	5	160.53
2032	6	57.48	2034	6	175.60	2036	6	157.19
2032	7	63.57	2034	7	176.46	2036	7	153.60
2032	8	70.01	2034	8	177.16	2036	8	149.79
2032	9	76.76	2034	9	177.72	2036	9	145.80
2032	10	83.76	2034	10	178.19	2036	10	141.65
2032	11	90.94	2034	11	178.56	2036	11	137.37
2032	12	98.21	2034	12	178.86	2036	12	133.02

Table S5. Monthly forecasted sunspot numbers for Solar Cycle 26 obtained using the ARFIMA model (continued).

Year	Month	Forecast	Year	Month	Forecast	Year	Month	Forecast
2037	1	128.62	2039	1	48.48	2040	7	14.18
2037	2	124.20	2039	2	46.04	2040	8	13.20
2037	3	119.80	2039	3	43.63	2040	9	12.33
2037	4	115.45	2039	4	41.25	2040	10	11.56
2037	5	111.17	2039	5	38.90	2040	11	10.89
2037	6	106.99	2039	6	36.60	2040	12	10.31
2037	7	102.91	2039	7	34.35	2041	1	9.82
2037	8	98.96	2039	8	32.16	2041	2	9.41
2037	9	95.14	2039	9	30.03	2041	3	9.09
2037	10	91.45	2039	10	27.99	2041	4	8.86
2037	11	87.90	2039	11	26.03	2041	5	8.70
2037	12	84.49	2039	12	24.17	2041	6	8.62
2038	1	81.21	2040	1	22.41	2041	7	8.62
2038	2	78.04	2040	2	20.76	2041	8	8.71
2038	3	75.00	2040	3	19.22	2041	9	8.88
2038	4	72.05	2040	4	17.79	2041	10	9.14
2038	5	69.20	2040	5	16.47	2041	11	9.51
2038	6	66.44	2040	6	15.27	2041	12	9.97
2038	7	63.74						
2038	8	61.09						
2038	9	58.50						
2038	10	55.95						
2038	11	53.43						
2038	12	50.95						
